# Translation and Validation of a Youth Self-Rated Insomnia Scale (YSIS) for Peruvian Adolescents

**DOI:** 10.3390/healthcare14080973

**Published:** 2026-04-08

**Authors:** Jessica J. Lucchini-Paredes, Alcides Flores-Paredes, Josue Pilco-Pezo, Gutember Peralta-Eugenio, Liset Z. Sairitupa-Sanchez, Sandra B. Morales-García, Oriana Rivera-Lozada, Patricia Soto-Casquero, Wilter C. Morales-García

**Affiliations:** 1 Unidad de Salud Pública, Escuela de Posgrado, Universidad Peruana Unión, Lima 15102, Peru; 2 Escuela Profesional de Educación Física, Universidad Nacional del Altiplano, Puno 21001, Peru; 3 Unidad de Administración, Escuela de Posgrado, Universidad Peruana Unión, Lima 15102, Peru; 4 Facultad de Ciencias de la Salud, Escuela de Psicología, Universidad Cesar Vallejo, Chimbote 02801, Peru; 5 Psicología, Universidad Señor de Sipán, Chiclayo 14001, Peru; 6 Medicina Humana, Universidad Señor de Sipán, Chiclayo 14001, Peruriveraoriana@uss.edu.pe (O.R.-L.); 7 Unidad de Psicología, Escuela de Posgrado, Universidad Peruana Unión, Lima 15102, Peru; 8 Dirección General de Investigación, Universidad Peruana Unión, Lima 15102, Peru; 9 Facultad de Teología, Universidad Peruana Unión, Lima 15102, Peru

**Keywords:** insomnia, adolescents, validation, psychometrics, Peru

## Abstract

**Background**: Adolescent insomnia is a public health concern associated with affective disturbances, poor academic performance, and cardiometabolic risk. In Peru, nighttime screen use, psychosocial stressors, and social inequalities intensify its impact; however, brief, validated screening instruments remain limited. **Objective**: To translate, culturally adapt, and evaluate the psychometric properties of the Youth Self-rated Insomnia Scale (YSIS) in Peruvian adolescents, examining its internal structure, reliability, and invariance across sex. **Methods**: An instrumental study was conducted with 300 students aged 13 to 17 years (M = 15.02; SD = 1.07). Descriptive statistics were calculated, and confirmatory factor analysis (CFA) was performed using a robust estimator. Reliability was assessed through Cronbach’s alpha (α), McDonald’s omega (ω), and average variance extracted (AVE). Factorial invariance by sex was examined at the configural, metric, scalar, and strict levels. **Results**: The unidimensional model demonstrated adequate fit (χ^2^ = 44.55, df = 18, *p* < 0.001; CFI = 0.97; TLI = 0.95; RMSEA = 0.07; SRMR = 0.04), with factor loadings ranging from 0.47 to 0.76, high internal consistency (α = 0.86; ω = 0.81), and AVE = 0.51. Although the two-factor model showed acceptable global fit indices, it revealed insufficient AVE in one factor (AVE = 0.40) and a high inter-factor correlation (r = 0.93), suggesting a lack of discriminant validity. Factorial invariance across sex was supported at all evaluated levels. **Conclusions**: The Spanish version of the YSIS demonstrates a unidimensional structure, adequate internal consistency, and factorial invariance across sex, supporting its use as a brief screening tool in clinical and school settings, as well as in epidemiological studies among Peruvian and Latin American adolescents.

## 1. Introduction

Insomnia in adolescents and young people has emerged as a public health problem with far-reaching effects on brain development, mental health, and academic performance, rather than a transient difficulty “falling asleep” [1]. Clinically, both the ICSD-3 and the DSM-5 define insomnia as persistent complaints of difficulty initiating sleep (DIS), difficulty maintaining sleep (DMS), or early morning awakening (EMA) despite adequate opportunity to sleep, accompanied by daytime impairment and a chronic course when it occurs ≥3 nights per week for ≥3 months—an approach that anchors diagnosis and prevents trivialization [2,3]. This definition is critical because, in adolescence, insomnia lies at the intersection of neurobiological maturation, psychosocial pressure, and digital lifestyles, shaping a high-risk phenotype that demands integrated explanations and solutions [4]. From a developmental perspective, adolescence reshapes sleep architecture: slow-wave sleep decreases, the tendency toward delayed circadian phases intensifies, and REM sleep latency shortens changes that collide with fixed school schedules and escalating academic and social demands [5,6,7,8]. This biologically vulnerable terrain overlaps with psychological and emotional factors that sustain a vicious cycle of a “hyper-alert mind” incompatible with sleep initiation and maintenance. Thus, identifying youth insomnia as a multifactorial disorder (biological, emotional, cognitive, and personality-related) is necessary for implementing effective and timely interventions [9]

Certain personality traits and styles of social evaluation amplify risk: self-oriented and socially prescribed perfectionism increases intolerance of “non-optimal sleep,” fuels repetitive thinking, and heightens autonomic activation mechanisms that appear particularly marked in females, for whom stress reactivity mediates insomnia severity [10]. In parallel, the nocturnal digital ecosystem, compulsive social media use, expectations of immediate responsiveness, and light exposure disrupt circadian rhythmicity and keep the cortex “switched on,” a pattern that perpetuates insomnia and undermines restorative sleep. Nomophobia, defined as anxiety about being disconnected, exacerbates this hypervigilance by sustaining a persistent pre-sleep alert state and consolidating technological dependence with adverse affective and attentional effects [11]. Somatic and lifestyle factors also contribute in non-trivial ways: high intake of ultra-processed foods has been associated with up to fourfold higher odds of insomnia among adolescent girls, even after adjustment for age, body mass index, and physical activity, suggesting metabolic and neurochemical pathways (e.g., glycemic instability, low-grade inflammation) that interfere with sleep regulation. This poorer diet also correlates with lower quality of life, underscoring that sleep hygiene should be paired with nutritional hygiene to achieve sustainable benefits [12].

At the core of the pathophysiology, evidence converges on hyperarousal: cognitive, affective, autonomic, and cortical overactivation that increases sleep onset latency, fragments continuity, and reduces sleep efficiency [13]. In adolescents, dysregulation of the autonomic nervous system and the hypothalamic–pituitary–adrenal axis maintains an “alertness signature” incompatible with nighttime shutdown, further intensified by academic stress and social pressure [14]. This helps explain why insomnia not only co-occurs with other mental health problems but may also precede and precipitate depression, anxiety, self-harm behaviors, and heightened suicide risk, a phenomenon intensified during the pandemic due to increased stress, isolation, and screen exposure [15]. Indeed, insomnia and evening preference are independently associated with greater sleepiness, depression, and anxiety; their combination increases daytime fatigue nearly ninefold and doubles the risk of suicidal ideation, even after controlling for affective symptoms [16]. Cognitive and academic consequences are similarly compelling: daytime sleepiness from insufficient sleep impairs sustained attention and performance in domains such as mathematics, with cumulative effects when sleep irregularity coexists with late chronotypes and early school start times [17,18,19]. At the same time, chronic insomnia is linked to long-term cardiometabolic risk trajectories, making early detection and treatment essential to alter its course [20,21]. In youth clinical populations, the burden of sleep disorders is substantial: about 40% report sleepiness and insomnia in major depression, and these disturbances are associated with poorer quality of life and greater suicidal ideation [22,23,24].

In this regard, adolescent insomnia represents a significant and growing problem that affects health and well-being in a comprehensive manner. This sleep disorder is associated with a wide range of negative outcomes, spanning academic impairment to mental health problems. Its prevalence in this population has increased over recent decades, with studies indicating that between 23% and 40% of adolescents experience sleep-related problems, an unusually high proportion compared with other life stages [1]. Adolescents with insomnia frequently report excessive daytime sleepiness, which has been shown to significantly affect academic performance, particularly in areas such as mathematics [25]. Moreover, insomnia is not only common and often chronic during this developmental period, but it also coexists with or precedes the onset of psychiatric disorders such as depression and anxiety, underscoring the need to address it seriously [16,26]. Contributing factors are multifaceted and include academic and social stress, electronic device use before bedtime, and puberty-related biological changes that disrupt normal sleep patterns [17,18]. These factors are often exacerbated by evening chronotypes, reflecting a preference for later activities that is associated with problems in daytime, emotional, and behavioral functioning, including suicide risk [16]. Insomnia and other sleep disorders, such as delayed sleep–wake phase disorder, are interrelated and share features that suggest the need for a broad and careful clinical approach to treatment and prevention [27]. Such an approach should include assessment and management of chronotype and promotion of adequate sleep hygiene practices, such as controlling exposure to blue light from electronic devices, which suppresses melatonin and may aggravate insomnia [17].

In Peru, insomnia has emerged as a particularly complex public health problem, exacerbated by socioenvironmental and psychological factors. During the COVID-19 pandemic, increased internet use and the consequent disruption of daily routines intensified this phenomenon. In Peru, approximately 65% of adults and one-third of adolescents face serious sleep difficulties, with a notably high prevalence of insomnia among young people [28]. Peruvian adolescents, in particular, show high rates of mental health problems such as anxiety and depression, partly influenced by prolonged electronic device use. Reports indicate that adolescents spend between 5 and 10 h per day in front of screens, which correlates with a 25.9% prevalence of internet addiction disorder (IAD). These stressors, combined with pandemic-related social isolation, have created fertile ground for insomnia, with reports indicating that a substantial 68.8% of adolescents experience depression and 27.7% anxiety [29]. Insomnia is not only associated with existing mental health problems but also acts as a risk factor for developing new disorders. It is well documented that sleep loss can lead to depressive and anxiety symptoms, especially under conditions of post-traumatic stress. In Latin America, factors such as education level and income also influence sleep quality, suggesting that lower socioeconomic status correlates with poorer sleep and a higher prevalence of insomnia [30]. Targeted interventions are therefore needed to address and mitigate insomnia’s impact on adolescents, particularly given its links not only to anxiety and depression but also to suicidal ideation (SI), a critical mental health warning sign in this population [31].

In the assessment of insomnia among adolescents, the availability and applicability of disorder-specific scales have been limited. Nevertheless, some tools have shown promise in this area. The EPR-A, which evaluates the relationship between subthreshold psychotic experiences, insomnia symptoms, resilience, and suicidal ideation, comprises 26 items across four dimensions: Delusional Experiences, Hallucinatory Experiences, Insomnia Symptoms, and Resilience. The scale uses a 5-point Likert format and has demonstrated high reliability, with Cronbach’s alpha ranging from 0.83 to 0.97 across subscales; it was validated in a sample of university students in China, providing a robust instrument for exploring these psychological phenomena in adolescents [32]. By contrast, the YSIS stands out as particularly suitable for adolescent populations. This 8-item scale is designed to assess insomnia severity over the past month. Although originally validated in a general adolescent population, it has shown particular utility in adolescents with psychiatric conditions, as demonstrated in a study conducted at the Shandong Mental Health Center. In that study, the YSIS not only correlated significantly with depression scores but was also associated with longer sleep onset latency, shorter sleep duration, and more frequent nightmares, supporting its construct and criterion-related validity. Internal consistency reliability was excellent, with an omega coefficient of 0.84, reaffirming its usefulness for this demographic group [33].

As a short 8-item measure, the YSIS offers a practical and accessible tool for rapid insomnia assessment. Its brevity is especially advantageous in clinical and educational settings where time and resources may be limited. In particular, implementing the YSIS provides a reliable and valid method to measure insomnia severity, thereby facilitating more effective and targeted interventions for this vulnerable group.

Therefore, the present study aims to evaluate the psychometric properties of the Youth Self-rated Insomnia Scale (YSIS) in a sample of Peruvian adolescents.

## 2. Materials and Methods

### 2.1. Study Design and Participants

An instrumental, cross-sectional study was conducted [34] to translate, culturally adapt, and evaluate the psychometric properties of the Youth Self-rated Insomnia Scale (YSIS) in Peruvian adolescents. A non-probabilistic convenience sampling approach was used. The minimum sample size was estimated using Soper’s calculator (2020), assuming a model with eight indicators, a simple structure, and a desired power of 1−β = 0.90 at α = 0.05, yielding a requirement of *n* ≥ 199. A total of 300 school-attending adolescents aged 13–17 years were recruited (M = 15.02; SD = 1.07). Most participants were male (51.0%), were 15 or 16 years old (both groups, 32.7%), and were enrolled in the fourth year of secondary school (42.7%), indicating a meaningful concentration in mid-adolescence and mid-level schooling (see Table 1).

### 2.2. Instruments

*Insomnia*. The English version of the Youth Self-Rating Insomnia Scale (YSIS) was used, an instrument designed to assess the severity of insomnia and its daytime consequences in adolescents. The scale consists of eight items organized into two dimensions: “Insomnia symptoms” (3 items) and “Daytime impairment or distress” (5 items). Each item is rated on a 5-point Likert-type scale. The scale’s reliability has been supported by a Cronbach’s alpha of 0.80 for the full scale and test–retest reliability of 0.82. The YSIS was validated in a sample of 11,626 adolescents in China using factor analyses that confirmed its two-factor structure. Although the scale has demonstrated validity and reliability in the Chinese population, it requires validation in clinical samples and other cultural contexts to ensure its applicability and accuracy across different adolescent groups [35].

The Spanish translation of the YSIS was conducted following internationally recognized guidelines for cross-cultural test adaptation, including the International Test Commission (ITC) Guidelines for Translating and Adapting Tests [36], as well as established methodological recommendations [37]. These procedures aim to ensure semantic, conceptual, and cultural equivalence between the original and adapted versions of the instrument.

1. Two bilingual translators (Spanish–English), both native Spanish speakers, independently produced the initial Spanish translation of the YSIS. The two versions were then compared, and a consensus version was developed.
2. This consensus version was back-translated into English by two native English speakers (from the United States) with proficiency in Spanish, who were unfamiliar with the instrument. This step aimed to verify fidelity to the original meaning.
3. An expert committee consisting of two educators and one psychologist reviewed the Spanish version along with the back-translated English versions in order to develop a preliminary Spanish version of the YSIS.
4. This preliminary version was administered to a focus group of 15 adolescents to assess comprehension, readability, and age-appropriate language. Based on their feedback, minor linguistic refinements were introduced to improve clarity.

During the expert committee review and cognitive testing phase, all items were carefully examined for semantic clarity, cultural appropriateness, and developmental comprehensibility. No substantive conceptual modifications were required, as the original item content was considered culturally applicable to Peruvian adolescents. The refinements introduced were limited to stylistic and linguistic clarity and did not alter the meaning or construct representation of any item. The final version was designated as the Youth Self-Rated Insomnia Scale—Short Spanish Form (YSIS-S), presented in Table 1.

### 2.3. Procedure

This study was conducted in accordance with rigorous ethical principles and was approved by the Ethics Committee of the Peruvian Union University (approval code: 2025-CE-EPG-00025). Data privacy and confidentiality were ensured, and informed consent was obtained from parents or legal guardians, along with adolescents’ informed assent, prior to instrument administration. Data collection was carried out in person at two educational institutions, emphasizing at all times the voluntary, confidential, and anonymous nature of participation. This methodological approach not only supports the validity and reliability of the data collected, but also ensures respect for and protection of participants’ rights throughout the research process.

### 2.4. Data Analysis

First, a descriptive item analysis of the YSIS-S was conducted, including measures of central tendency and dispersion (mean and standard deviation), as well as skewness (g_1_) and kurtosis (g_2_) indices. Skewness and kurtosis values within ±2.0 were considered aceptable [38]. In addition, corrected item–total correlations were examined to identify and exclude items with coefficients ≤ 0.30 or evidence of multicollinearity [39].

Next, confirmatory factor analysis (CFA) was performed to evaluate the scale’s one-factor structure using the Weighted Least Squares Mean and Variance adjusted (WLSMV) estimator, which is recommended for ordinal Likert-type data and does not assume multivariate normality [40]. Model fit was evaluated using: chi-square (χ^2^), the Comparative Fit Index (CFI), and the Tucker–Lewis Index (TLI), considering values ≥ 0.95 as indicative of good fit, as well as the Root Mean Square Error of Approximation (RMSEA) and the Standardized Root Mean Square Residual (SRMR), considering values ≤ 0.08 [39,41]. Internal consistency reliability was assessed using Cronbach’s alpha and McDonald’s omega, with values > 0.70 considered adequate [42].

To examine measurement invariance (MI), multigroup CFA was conducted to evaluate whether the factorial structure of the YSIS-S was equivalent across relevant demographic groups. Given prior evidence suggesting sex-related differences in insomnia symptomatology during adolescence, sex-based invariance was tested. Configural, metric, scalar, and strict invariance were sequentially evaluated using the WLSMV estimator, appropriate for ordinal data. Invariance was considered supported when ΔCFI ≤ 0.010, ΔRMSEA ≤ 0.015, and ΔSRMR ≤ 0.030 at the metric step and ≤0.010 at the scalar step [43]. Additionally, an explanatory model was tested using structural equation modeling, applying the WLSMV estimator and the same fit criteria described above.

All statistical analyses were performed in Posit Software (2024) (RStudio, Version 2024.04.2+764; http://www.posit.co/) using R version 4.1.1 (R Foundation for Statistical Computing, Vienna, Austria; http://www.R-project.org). Confirmatory factor analyses and structural modeling were conducted with the lavaan package [44], while measurement invariance analyses were supported using the semTools package [45].

## 3. Results

### 3.1. Item Descriptive Statistics

Descriptive analyses showed that YSIS-S item means ranged from 2.47 to 3.19. Item 7 had the highest mean (M = 3.19), indicating that participants reported the greatest frequency or intensity for this symptom. In contrast, item 5 had the lowest mean (M = 2.47), suggesting that it was the least problematic aspect perceived by respondents. Regarding item distributions, skewness (g_1_) and kurtosis (g_2_) values fell within the acceptable ±1.5 range, indicating approximately normal distributions for each item. This supports the suitability of the data for parametric techniques such as CFA. For corrected item–total correlations (r.cor), all items exceeded the 0.30 threshold, ranging from 0.53 (items 1 and 2) to 0.68 (item 3). These findings indicate that all items contribute meaningfully to the general construct measured by the scale, with no evidence suggesting item removal due to poor psychometric performance. In particular, items 3, 6, 7, and 8 showed the strongest item–total associations, reflecting a closer relationship with the overall insomnia construct (Table 2).

### 3.2. Internal Structure and Reliability

A confirmatory factor analysis (CFA) was conducted to evaluate the internal structure of the YSIS-S. First, a two correlated-factor model (Model 1) was estimated, in which items were grouped into Daytime Distress (items 1, 2, 6, 7, and 8) and Insomnia Symptoms (items 3, 4, and 5). However, the overall fit of the bifactorial model was inadequate: χ^2^ = 377.990, df = 19, *p* < 0.001; CFI = 0.86; TLI = 0.79; RMSEA = 0.25 (90% CI [0.23, 0.27]); SRMR = 0.12. The CFI and TLI values fall below the recommended cutoff (0.90–0.95), while the RMSEA (0.25) and SRMR (0.12) substantially exceed acceptable thresholds (<0.08), indicating clearly poor model fit. Although standardized factor loadings were high (ranging from 0.70 to 0.82) and convergent validity was adequate (AVE = 0.60 for Daytime Distress and AVE = 0.57 for Insomnia Symptoms), the inter-factor correlation was high (r = 0.81). Applying the Fornell–Larcker criterion, the shared variance between factors (r^2^ = 0.66) exceeded the AVE values of both constructs, indicating a lack of discriminant validity. Overall, these findings suggest that, despite internal coherence within factors, the bifactorial model does not adequately represent the empirical structure of the instrument. Subsequently, a unidimensional model (Model 2) was estimated, in which all eight items loaded onto a single factor. This model demonstrated substantially better fit: χ^2^ = 48.730, df = 17, *p* < 0.001; CFI = 0.99; TLI = 0.98; RMSEA = 0.08 (90% CI [0.05, 0.11]); SRMR = 0.04. The CFI and TLI values exceed the 0.95 criterion, the SRMR remains below 0.05, and the RMSEA is at the upper limit of acceptability, supporting an overall adequate fit. Factor loadings ranged from 0.51 to 0.81, with items 1 and 2 showing the lowest loadings, though still within acceptable ranges. The internal consistency of the unidimensional model was high (α = 0.86; ω = 0.81), indicating adequate reliability. Taken together, the results indicate that the unidimensional model provides a substantially better fit than the bifactorial model and avoids issues of discriminant validity. Therefore, the empirical evidence supports the unidimensional structure as the most parsimonious and psychometrically robust representation for measuring insomnia in adolescents using the YSIS-S (Table 3).

### 3.3. Invariance

The hierarchical evaluation of the factorial invariance of the YSIS-S by sex was conducted using multigroup confirmatory factor analysis. The results demonstrated adequate retention at all levels of invariance: configural, metric, scalar, and strict. The configural model showed acceptable fit (χ^2^ = 53.54, df = 34, *p* = 0.018; CFI = 0.99; TLI = 0.942; RMSEA = 0.062; SRMR = 0.040), indicating that the factorial structure is equivalent across females and males. When equality constraints were imposed on the factor loadings (metric model), model fit did not deteriorate (χ^2^ = 40.884, df = 41, *p* = 0.476; CFI = 1.00; TLI = 1.00; RMSEA = 0.000; SRMR = 0.042). Changes relative to the configural model remained within recommended thresholds (ΔCFI = 0.010; ΔRMSEA = −0.062; ΔSRMR = 0.002), supporting the equivalence of factor loadings across groups. At the scalar level, after constraining item intercepts, the model continued to show excellent fit (χ^2^ = 45.77, df = 48, *p* = 0.565; CFI = 1.00; TLI = 1.00; RMSEA = 0.000; SRMR = 0.044), with minimal changes relative to the metric model (ΔCFI = 0.000; ΔRMSEA = 0.000; ΔSRMR = +0.002), indicating invariant intercepts. Finally, the strict model also demonstrated adequate fit (χ^2^ = 53.238, df = 56, *p* = 0.580; CFI = 1.00; TLI = 1.00; RMSEA = 0.000; SRMR = 0.049), with no meaningful deterioration compared to the scalar model (ΔCFI = 0.000; ΔRMSEA = 0.000; ΔSRMR = 0.005), supporting the equivalence of measurement errors. Overall, the findings confirm full factorial invariance of the YSIS-S across sex. This implies that the instrument measures the construct of insomnia equivalently in females and males, allowing for valid comparisons of both latent means and structural relationships between groups (Table 4).

## 4. Discussion

The present study aimed to evaluate the internal structure and psychometric properties of the YSIS-S in Peruvian adolescents, given the growing relevance of youth insomnia as a public health concern. The literature has documented that insomnia during adolescence reflects a complex interaction among maturational changes in the circadian system, increased vulnerability to cognitive and emotional hyperarousal, and psychosocial stressors amplified by nighttime use of electronic devices [5,13]. This profile is consistently associated with impaired daytime functioning, poor academic performance, and greater affective symptomatology [16], underscoring the need for brief, valid, and culturally adapted instruments for early detection in school settings. From a structural standpoint, our findings did not support the bifactorial model originally proposed in other international validations. Although factor loadings were high and convergent validity was adequate for both factors, the two-dimensional model demonstrated clearly inadequate overall fit (CFI = 0.86; TLI = 0.79; RMSEA = 0.25; SRMR = 0.12). In addition, the inter-factor correlation was high (r = 0.81), and the Fornell–Larcker analysis indicated a lack of discriminant validity, as the shared variance between factors exceeded the average variance extracted for each. These results suggest substantial conceptual overlap between nighttime symptoms and daytime distress, calling into question the empirical distinction between these dimensions in this population. In contrast, the unidimensional model showed substantially superior fit (CFI = 0.99; TLI = 0.98; RMSEA = 0.08; SRMR = 0.04), along with adequate factor loadings and high internal consistency (α = 0.86; ω = 0.81). Although the RMSEA was at the upper limit of acceptability, the remaining indices indicated excellent fit, and the model demonstrated greater parsimony and conceptual coherence. From a psychometric perspective, these findings support interpreting adolescent insomnia as a unitary construct in the general population, in which nighttime symptoms and daytime impairment are experienced as an integrated dimension of sleep-related distress rather than as clearly differentiated domains. This pattern may be explained by the developmental nature of insomnia in non-clinical adolescents. Unlike clinical samples, where symptoms may organize into more distinct domains, in school-based populations nighttime complaints and daytime impairment tend to co-occur as a global experience of sleep disturbance. Thus, the unidimensional structure is not only statistically more robust but also conceptually aligned with how adolescents perceive and report their sleep difficulties.

Regarding reliability, the obtained coefficients exceeded conventional standards for applied research, supporting the use of the total score as a continuous global indicator of insomnia severity. The slight difference between alpha and omega may be attributed to the lack of strict equivalence in factor loadings; however, this does not compromise the internal stability of the instrument.

A relevant contribution of this study is the demonstration of full factorial invariance across sex. Multigroup analysis supported configural, metric, scalar, and strict invariance, with no significant deterioration in fit across increasingly constrained models. This indicates that the YSIS-S measures the construct of insomnia equivalently in females and males, allowing for valid comparisons of both latent means and structural relationships. From an applied perspective, this finding reduces the risk of measurement bias and strengthens its utility in epidemiological surveillance, school-based assessment, and sex-based comparative studies.

Although age-related group comparisons were not a predefined objective of this study, adolescence is characterized by developmental changes in sleep regulation (e.g., circadian phase delay and increased vulnerability to sleep restriction) that may influence perceived insomnia severity. Future studies with larger and more balanced age distributions should examine potential age-related trends in YSIS-S total scores and evaluate developmental measurement properties when appropriate [5,13].

Overall, the empirical evidence indicates that the Spanish version of the YSIS-S demonstrates a parsimonious unidimensional structure, adequate internal consistency, and metric equivalence across sex. These results support its use as a brief screening tool for Peruvian adolescents and contribute to addressing a methodological gap in the assessment of youth insomnia in Latin American contexts.

### 4.1. Implications

The validation of the YSIS-S in Peruvian adolescents represents a meaningful methodological advancement for insomnia screening in Spanish-speaking contexts. Empirical evidence supporting a unidimensional structure, together with adequate internal consistency and solid indicators of internal structural validity, supports interpreting the total score as a continuous global index of insomnia severity. However, empirically derived cutoff scores and normative benchmarks have not yet been established and require future research to enhance clinical interpretability. Therefore, the YSIS-S should currently be interpreted as a dimensional screener rather than a diagnostic instrument with categorical decision thresholds. This parsimonious solution not only demonstrated better fit than the bifactorial model but also avoided problems of discriminant validity stemming from the high inter-factor correlation observed in the two-factor solution. From a theoretical perspective, the findings support integrative models of adolescent insomnia, suggesting that nighttime difficulties and daytime distress represent manifestations of a unitary construct in general school-based populations. This is consistent with dimensional approaches to insomnia that emphasize continuity between subjective sleep experience and daytime functioning. At the applied level, the demonstration of full factorial invariance by sex (configural, metric, scalar, and strict) ensures that the instrument measures the con-struct equivalently in females and males. Consequently, valid comparisons of both observed scores and latent means across sex are possible, reducing the risk of measurement bias in comparative research and clinical decision-making. For educational and healthcare systems, the YSIS-S offers a brief, comprehensible, and low-cost tool that can facilitate school-based screening, early identification of more severe cases, and timely referral to evidence-based interventions, such as cognitive behavioral therapy for insomnia (CBT-I), sleep hygiene programs, and strategies to regulate nighttime screen use. At the public health level, its standardized application could contribute to strengthening epidemiological surveillance of adolescent sleep and to planning targeted preventive interventions.

### 4.2. Limitations

This study has several limitations that should be considered when interpreting the findings. First, the cross-sectional design precludes examination of temporal stability, test–retest reliability, sensitivity to change, and longitudinal measurement invariance. Temporal stability is particularly relevant for screening instruments; therefore, future research should incorporate prospective designs and retest subsamples after short intervals (e.g., 2–4 weeks) to estimate intraclass correlation coefficients and evaluate score consistency over time.

Second, although the sample size met minimum recommended criteria for confirmatory factor analysis with eight indicators, moderate sample sizes may introduce some instability in parameter estimates, particularly in standard errors and the precision of factor loadings, especially when multigroup models are employed. Studies with larger samples would yield more stable and robust estimates, reducing potential variability in structural parameters. Additionally, the sampling strategy was non-probabilistic and limited to a small number of educational institutions, which restricts generalizability to other regions or sociocultural contexts within the country. A notable concentration of participants in certain grade levels (particularly the fourth year of secondary school) was also observed, potentially introducing structural bias related to developmental and academic stages. Because sleep characteristics may vary by educational level and academic demands, this imbalance could have influenced the estimated factorial structure. Future research should incorporate probabilistic sampling and a more balanced distribution across educational levels to examine the stability of the model throughout different stages of adolescence. Although age-related comparisons were discussed conceptually, measurement invariance across age groups was not included as a predefined analytical objective due to uneven subgroup distribution and the primary focus on structural validation. Future studies with larger and developmentally balanced samples should evaluate age-related score differences and developmental measurement properties more rigorously.

Third, assessment relied exclusively on self-report measures, which may introduce biases related to social desirability or retrospective recall. Triangulation with objective measures (e.g., actigraphy), structured clinical interviews, or parental reports would strengthen the validity evidence. External criterion and convergent validity were not examined through associations with related variables such as daytime sleepiness, chronotype, affective symptomatology, academic performance, or clinical insomnia diagnoses. The absence of external validation indicators limits the breadth of construct validity evidence provided in the present study. Future research should incorporate theoretically related constructs and clinical benchmarks to establish convergent, discriminant, and criterion validity. Empirically grounded cutoff scores and normative benchmarks were also not established, which limits immediate clinical interpretability. Future investigations should determine optimal cut points using receiver operating characteristic (ROC) analyses against diagnostic criteria or clinical interviews to enhance screening precision.

Finally, although confirmatory factor analysis was estimated using an estimator appropriate for ordinal data, replication in independent samples and diverse cultural contexts would further strengthen the stability, cross-cultural generalizability, and interpretative robustness of the proposed unidimensional solution.

## 5. Conclusions

The Spanish version of the YSIS-S demonstrates solid evidence of internal structural validity based on confirmatory factor analysis and reliability indices and full factorial invariance across sex in Peruvian adolescents. The findings support a parsimonious unidimensional structure, allowing the total score to be interpreted as a continuous indicator of insomnia severity rather than as a categorical diagnostic classification. Collectively, these results endorse the YSIS-S as a brief and psychometrically sound tool for clinical and school-based screening, group comparisons, and epidemiological studies in adolescent populations. Nevertheless, further research is needed to extend validation to clinical samples; establish external criterion validity and empirically supported cutoff scores; and evaluate temporal stability and sensitivity to change following evidence-based interventions in order to consolidate its diagnostic utility and public health contribution.

## Figures and Tables

**Table 1:** Sociodemographic Characteristics.

Characteristics		*n*	%
Sex	Female	147	49.0
	Male	153	51.0
Age	13	33	11.0
	14	56	18.7
	15	98	32.7
	16	98	32.7
	17	15	5.0
School Year	First	20	6.7
	Second	57	19
	Third	57	19
	Fourth	128	42.7
	Fifth	38	12.7

**Table 2:** Descriptive Statistics.

Item in English	Item in Spanish	M	sd	g_1_	g_2_	r.cor
1. During the past month, how would you rate the quality of your sleep overall?	Durante el último mes, ¿cómo calificarías la calidad de tu sueño en general?	2.76	1.13	0.12	−0.63	0.53
2. During the past month, how satisfied were you with your sleep overall?	Durante el último mes, ¿cuán satisfecho/a estuviste con tu sueño en general?	2.77	1.13	0.02	−0.74	0.53
3. Trouble falling asleep	Dificultad para conciliar el sueño	2.62	1.24	0.36	−0.81	0.68
4. Wake up frequently during the night	Despertar frecuentemente durante la noche	2.51	1.22	0.46	−0.65	0.61
5. Wake up very early and can’t get back to sleep	Despertar muy temprano y no poder volver a dormir	2.47	1.21	0.38	−0.77	0.55
6. Do not have enough sleep	No tener suficiente sueño	2.64	1.2	0.28	−0.78	0.67
7. Feel unrested and unrestored upon waking	Sentirse cansado/a y sin energía al despertar	3.19	1.25	−0.13	−0.99	0.64
8. Sleep disturbance interferes with your daily activities	La alteración del sueño interfiere con tus actividades diarias	2.89	1.16	.14	−0.68	0.64

Notes: M = mean; sd = standard deviation; g_1_ = skewness; g_2_ = kurtosis; r.cor = corrected item–total correlation.

**Table 3:** Confirmatory Factor Analysis and Reliability.

Item	Model 1		Model 2 (λ)
	F1(λ)	F2(λ)	
1	0.78	—	0.51
2	0.79	—	0.51
3	—	0.82	0.79
4	—	0.74	0.69
5	—	0.7	0.65
6	0.76	—	0.81
7	0.78	—	0.72
8	0.76	—	0.69
α	0.81	0.76	0.86
ω	0.86	0.77	0.81
AVE	0.6	0.57	—
r (F1, F2)	0.81		
r^2^ (F1, F2)	0.66		—

Notes: Standardized loadings (λ). r (F1, F2): inter-factor correlation in Model 1. r^2^ = squared inter-factor correlation. AVE: Average Variance Extracted.

**Table 4:** Measurement Invariance by Sex.

Model	χ^2^	gl	p	CFI	TLI	RMSEA	SRMR	ΔCFI	ΔRMSEA	ΔSRMR
Configural	53.54	34	0.018	0.99	0.942	0.062	0.04	—	—	—
Metric	40.884	41	0.476	1.000	1.000	0.000	0.042	0.010	−0.062	0.002
Scalar	45.77	48	0.565	1.000	1.000	0.000	0.044	0.000	0.000	0.002
Strict	53.238	56	0.58	1.000	1.000	0.000	0.049	0.000	0.000	0.005

Notes: Δ indicates change relative to the immediately preceding model. Invariance evaluation was primarily based on changes in CFI, RMSEA, and SRMR, following Chen (2007) [43], given the use of the robust WLSMV estimator. The following criteria were considered acceptable: ΔCFI ≤ 0.010, ΔRMSEA ≤ 0.015, and ΔSRMR ≤ 0.030 (metric), ≤0.010 (scalar).

## Data Availability

The data presented in this study are available from the corresponding author upon reasonable request. The data are not publicly available due to ethical and privacy restrictions involving adolescent participants.
